# Strategic approaches in oral squamous cell carcinoma diagnostics using liquid biopsy

**DOI:** 10.1111/prd.12567

**Published:** 2024-04-27

**Authors:** Denis F. Kinane, Joerg Gabert, George Xynopoulos, Esra Guzeldemir‐Akcakanat

**Affiliations:** ^1^ Department of Periodontology, Dental School University Bern Bern Switzerland; ^2^ University of Leipzig Leipzig Germany; ^3^ Medical Diagnostics Ltd London UK; ^4^ Department of Periodontology, Faculty of Dentistry Kocaeli University İzmit Turkey; ^5^ College of Dental Medicine QU Health, Qatar University Qatar Qatar; ^6^ ExpressTest Cignpost Diagnostics Ltd. Farnborough United Kingdom

**Keywords:** liquid biopsy, next generation sequencing, oral squamous cell carcinoma

## Abstract

Liquid biopsy is a noninvasive diagnostic technique used for monitoring cancer utilizing specific genetic biomarkers present in bodily fluids, such as blood, saliva, or urine. These analyses employ multiple biomolecular sources including circulating tumor DNA (ctDNA), circulating tumor cells (CTCs), and exosomes (that contain DNA fragments) to detect genetic biomarkers that can predict, disclose, and/or monitor cancers. Levels of these biomarkers can inform on the presence of cancer, its genetic characteristics, and its potential treatment response and also provide predictive genetic predisposition information for specific cancers including oral squamous cell carcinomas (OSCC). Liquid biopsies can aid cancer management as they offer real‐time dynamic information on the response to say chemotherapy or radiotherapy and recurrence following surgical excision. Unlike traditional tissue biopsies, which are invasive with a degree of morbidity and require specific tumor location sampling, liquid biopsies are noninvasive and can be repeated frequently. For oral squamous cell carcinoma, on which this review focuses, liquid biopsy of blood or saliva can be valuable in predicting susceptibility, providing early detection, and monitoring the disease's progression and response to therapy. This review gives a general narrative overview of the technology, its current medical usage, and advantages and disadvantages compared with current techniques and discusses a range of current potential biomarkers for disclosing OSCC and predicting its risk. Oral squamous cell carcinoma is all too often detected in the late stages. In future, liquid biopsy may provide an effective screening process such that cancers including OSCC will be detected in the early stages rather than later when prognosis is poor and morbidity and debilitation are greater. In this screening process, periodontists and hygienists have a critical role in that they are adept in examining mucosa, they see patients with shared risk factors for periodontitis and OSCC, namely smoking and poor oral hygiene, and they see patients frequently such that OSCC examinations should be a routine part of the recall visit. With this additional screening manpower, oral medicine and oral surgery colleagues will detect OSCC earlier and this coupled with new techniques such as liquid biopsy may greatly decrease global morbidity in OSCC.

## 1 RISK FACTORS FOR ORAL CANCER AND THE PERIODONTISTS ROLE

### 1.1 Environmental risk factors for OSCC

Oral cancer has various risk factors that increase the likelihood of its development. Prior to discussing the utility of gene markers of susceptibility for oral cancer, it is important to bear in mind that any susceptibility is a combination of environmental and genetic factors. In OSCC, environmental factors such as smoking and betel nut use play a large part, whereas in other conditions, such as breast cancer hereditary elements may have a much bigger say in overall susceptibility. It is important to note that having one or more risk factors does not guarantee the development of oral cancer, but they can contribute to an increased risk.

Environmental factors include:

Tobacco use: smoking cigarettes, cigars, or pipes and using smokeless tobacco (e.g., chewing tobacco) are significant risk factors for oral cancer. Tobacco contains harmful chemicals that can damage the cells in the oral cavity and lead to cancer development.^1^
Alcohol consumption: Heavy and frequent alcohol consumption is associated with an increased risk of oral cancer. Alcohol can damage the cells lining the oral cavity and make them more susceptible to cancerous changes.^2^, ^3^
Betel quid chewing: The habit of chewing betel quid, especially when combined with tobacco and/or areca nut, is a significant risk factor for oral cancer in certain regions of the world.^4^
Human Papillomavirus (HPV) Infection: Certain strains of HPV, especially HPV16 and HPV18, have been linked to an increased risk of oral cancer, particularly in the oropharyngeal region.^5^

These risk factors can interact and compound each other, increasing the overall risk of developing oral cancer. Additionally, maintaining good oral hygiene and seeking regular dental checkups can help with early detection and prevention of oral cancer.

### 1.2 Genetic risk factors and potential biomarkers for OSCC

Oral squamous cell carcinoma (OSCC) is a type of cancer that arises from the squamous cells lining the oral cavity. Like many cancers, OSCC can be influenced by genetic risk factors. These factors are easily identified by NGS but it is essential to distinguish between risk factors that merely indicate an increased susceptibility to a particular disease and NGS markers of the actual tumor itself which disclose the actual presence of the cancer in question.

- TP53 gene mutations: The TP53 gene encodes the tumor suppressor protein p53, which plays a crucial role in cell cycle regulation and DNA repair. Mutations in the TP53 gene are frequently observed in OSCC and are associated with a higher risk of tumor development and progression. The TP53 gene is responsible for regulating cell division and preventing the formation of tumors. Mutations can lead to uncontrolled cell growth and an increased risk of tumor development.^6^ TP53 mutations may be both a risk element and a disclosing factor in OSCC.
- EGFR (epidermal growth factor receptor) alterations: EGFR is a receptor that regulates cell growth and division. Mutations in the EGFR gene can lead to abnormal cell proliferation and contribute to oral cancer development. Overexpression has been linked to OSCC development and aggressive tumor behavior. EGFR alterations may promote cell proliferation and resistance to apoptosis.^7^ EFGR mutations may be both a risk element and a disclosing factor in OSCC.
- Cyclin D1 (CCND1) gene amplification: Cyclin D1 is a regulatory protein that controls cell cycle progression. Amplification of the CCND1 gene has been found in OSCC and may contribute to uncontrolled cell growth.^8^ This CCND1 gene may be a marker of OSCC disease.
- CDKN2A (p16): This gene encodes a protein known as p16, which is also involved in cell cycle regulation. Alterations in CDKN2A, such as deletions or promoter hypermethylation, can lead to the loss of p16INK4a protein function, promoting OSCC development. The CDKN2A gene produces proteins that regulate the cell cycle, and alterations in this gene have been linked to a higher risk of oral cancer. These alterations can disrupt the normal cell cycle control, leading to uncontrolled cell growth.^9^ CDKN2A alterations may be both a risk element and a disclosing factor in OSCC.
- MTHFR (methylenetetrahydrofolate reductase) gene polymorphisms: MTHFR is involved in folate metabolism and DNA methylation. Polymorphisms in the MTHFR gene have been associated with an increased risk of OSCC, possibly due to alterations in DNA methylation patterns.^10^ MTHFR polymorphisms are most likely to be a risk‐invoking factor in OSCC.
- GSTM1 (Glutathione S‐transferase M1) and GSTT1 (Glutathione S‐transferase T1) gene deletions: Glutathione S‐transferases are involved in detoxification processes and cellular defense against oxidative stress. Deletions in GSTM1 and GSTT1 genes have been linked to OSCC susceptibility, as they may reduce the body's ability to metabolize carcinogens effectively^11^ and thus these deletions can be seen more as risk markers than biomarkers disclosing the presence of OSCC.
- CYP1A1 and CYP2E1: These genes encode enzymes involved in the metabolism of carcinogens found in tobacco and alcohol, both of which are significant risk factors for oral cancer. Variations in these genes might influence an individual's susceptibility to these carcinogens. Certain CYP1A1 gene polymorphisms have been associated with an increased risk of oral cancer due to altered enzyme activity and potential difficulties in detoxifying harmful substances, specifically, tobacco products^12^ and these are seen as risk factors rather than biomarkers of OSCC.
- XRCC1: This gene is involved in DNA repair processes. Certain variants of XRCC1 may affect an individual's ability to repair damaged DNA, potentially increasing the risk of cancer development, including oral cancer, again these mutations are more risk elements than biomarkers.
- ALDH2 Gene Polymorphism: Aldehyde dehydrogenase 2 (ALDH2) is an enzyme involved in alcohol metabolism. A specific polymorphism (ALDH2*2) results in reduced enzymatic activity and has been associated with an increased risk of oral cancer, especially in individuals who consume alcohol.^13^ Clearly, a risk factor linked with the other risk factor is alcohol consumption.

### 1.3 Oral cancer diagnostic procedures and the role of the periodontist

Diagnosing oral cancer involves a combination of clinical examination, imaging tests, and biopsies, these include the following procedures:

#### 1.3.1 Clinical examination

The first step in diagnosing oral cancer involves a thorough physical examination of the oral cavity. A dentist or oral healthcare professional will inspect the lips, tongue, gums, cheeks, roof, and floor of the mouth, looking for any abnormal areas, such as lumps, ulcers, or white or red patches.^14^, ^15^, ^16^ Periodontists and hygienists have a critical role to play in screening for OSCC as they see patients that share risk factors for periodontitis and OSCC, namely smoking and poor oral hygiene and in certain countries betel nut use. They are adept at examining soft tissue and assessing abnormal tissue and they see patients frequently such that OSCC screening should be a critical element of the recall appointment. Training and clinical experience are of course necessary in manipulating and assessing the tongue and palate and cheeks as well as looking at radiographs for abnormal masses but additionally, a strong referral network and rapport with oral medicine and oral surgery colleagues is invaluable as the diagnosis is complex and biopsies and subsequent management need to be carefully considered and performed.

#### 1.3.2 Imaging tests

Imaging tests can provide valuable information about the extent and spread of oral cancer. To further evaluate the extent of the disease and detect potential metastasis, imaging tests are commonly used. The most common imaging techniques include:

- X‐rays: Panoramic radiographs or intraoral X‐rays can help identify changes in bone structure and detect suspicious masses.
- Computed tomography (CT) scan: CT scans can provide detailed cross‐sectional images of the oral and maxillofacial regions, helping assess tumor size, invasion into nearby structures, and lymph node involvement.^15^
- Magnetic resonance imaging (MRI): MRI can provide detailed soft tissue images, useful for evaluating tumor extent and invasion into surrounding tissues.
- PET scan or positron emission tomography: PET scans use a radioactive tracer to detect areas with high metabolic activity, which can be indicative of cancerous tissue.^15^

#### 1.3.3 Surgical biopsy

The definitive diagnosis of oral cancer relies on a biopsy, which involves the removal of a small sample of suspicious tissue for examination under a microscope. There are different types of biopsies:

- Incisional biopsy and cytopathology: A small portion of the suspicious lesion is removed for examination.^16^
- Excisional biopsy: The entire lesion is removed if it is small enough, providing a more comprehensive sample for diagnosis.
- Fine‐needle aspiration (FNA) biopsy: Used for evaluating enlarged lymph nodes or distant metastases, a thin needle is inserted into the target area to collect cells for examination.^17^

## 2 THE LIQUID BIOPSY PROCESS

### 2.1 Liquid biopsy and next‐generation sequencing in cancer diagnostics

Liquid biopsy is a noninvasive diagnostic approach that involves the analysis of various biomolecules, such as circulating tumor DNA (ctDNA), circulating tumor cells (CTCs), exosomes (that contain DNA fragments), and other biomarkers, in blood or other bodily fluids to detect, predict, and monitor cancers. It has gained significant attention as a potential tool for early cancer detection, monitoring treatment response, and identifying genetic mutations in various types of cancers. The availability and accuracy of liquid biopsy tests can vary depending on the cancer type and the stage of the disease. As technology and understanding of cancer biology improve, liquid biopsy's applications will expand further and into different cancer systems such as OSCC, to which this review is dedicated. Liquid biopsy can be particularly useful in situations where traditional tissue biopsies are challenging, and it can also be used for monitoring treatment responses and detecting cancer recurrence in a minimally invasive manner. In the future, liquid biopsy may provide an effective screening process such that cancers will be detected in primary stages rather than in later stages where treatment is less successful and involves greater morbidity.

### 2.2 Sources of genetic material

**FIGURE 1:**
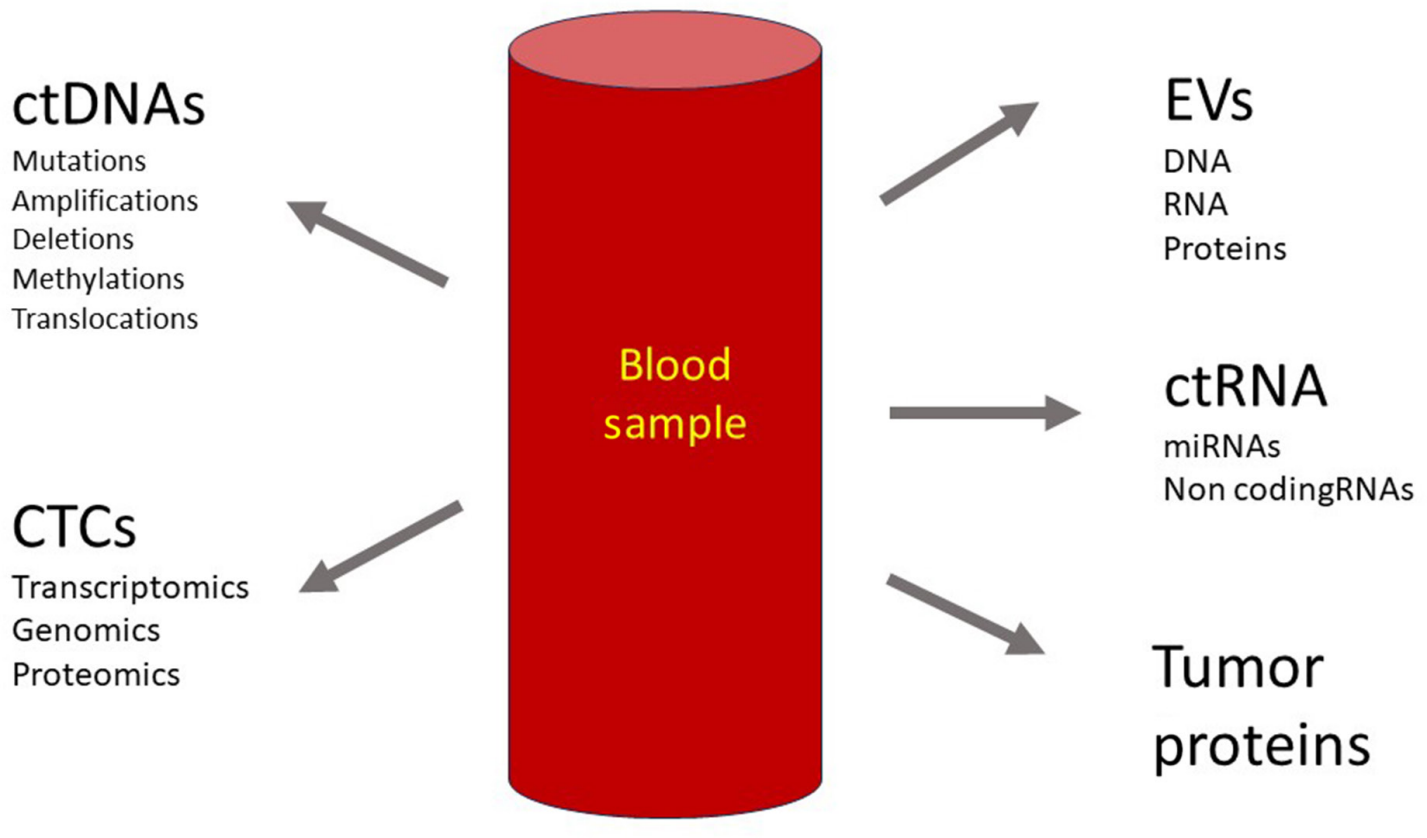
The peripheral blood sample is the major source of tumor‐related biomolecules for the liquid biopsy process and cancer diagnostics. Circulating tumor cells (CTCs), provide a rich source of tumor DNA, RNA, and proteins. Clearly, the next‐generation sequencing analysis will focus on the diagnostic genetic information but these cells provide much more and can be cultured ex vivo to provide a continuous source of relevant material and an opportunity to test anticancer molecules ex vivo. In a similar way, the constituents of extracellular vesicles (EVs) provide DNA, RNA, and protein. The blood sample also includes circulating tumor proteins and circulating tumor (ct)RNAs and DNAs. The information detectable from ctDNA includes; mutations, deletions, gene amplifications, methylation patterns, and translocations. miRNA expression panels and long noncoding (lnc)RNA expression are good sources of quantitative biomarker information.

Next‐generation sequencing (NGS): NGS technology allows high‐throughput sequencing of DNA and RNA, enabling the detection of genetic mutations, copy number variations, and other molecular alterations in ctDNA and other liquid biopsy analytes.^18^, ^19^ Figure 1 depicts the basic categories of biological material that can be obtained from a blood sample of a cancer patient that might be used for diagnosis.

Circulating tumor DNA (ctDNA) analysis: ctDNA refers to fragmented DNA shed by tumor cells into the bloodstream. ctDNA carries genetic information specific to the tumor and can be isolated and analyzed to detect cancer‐associated mutations, genomic alterations, and other molecular changes.^20^, ^21^

Circulating tumor cells (CTCs) analysis: CTCs are cancer cells that have detached from the primary tumor and entered the bloodstream. Isolation and analysis of CTCs can provide insights into tumor metastasis, disease progression, and treatment response.^22^, ^23^, ^24^ Studies have shown that ctDNA analysis can detect pancreatic cancer mutations and provide insights into tumor heterogeneity and evolution.^25^

Extracellular vesicle (EVs) analysis: EVs, such as exosomes, are small membrane‐bound vesicles released by cancer cells, carrying various biomolecules, including DNA, RNA, and proteins. Liquid biopsy can isolate and analyze EVs to identify, for example, specific pancreatic cancer‐associated markers.^26^ Exosomes reflect the molecular characteristics of the parent tumor.^27^, ^28^, ^29^

MicroRNA profiling: MicroRNAs (miRNAs) are small noncoding RNA molecules involved in the regulation of gene expression. Liquid biopsy can analyze miRNA profiles to identify unique patterns associated with tumors such as pancreatic cancer^30^ and OSCC.^31^

### 2.3 The liquid biopsy procedure

- Sample collection: A blood sample or other bodily fluid (such as urine, cerebrospinal fluid, or saliva) is collected from the patient. Blood is the most common fluid used for liquid biopsy due to its accessibility and the abundance of circulating tumor‐derived materials.
- Sample processing: The collected sample is processed to separate the plasma or serum from the blood cells. Various methods can be employed for plasma or serum isolation, including centrifugation and specialized kits.
- Isolation of circulating tumor biomarkers: ctDNA, circulating tumor cells (CTCs), exosomes, or other tumor‐specific biomarkers are isolated from the plasma or serum. Various techniques, such as polymerase chain reaction (PCR), digital PCR (dPCR), next‐generation sequencing (NGS), and microfluidics‐based platforms, can be used for this purpose.
- Biomarker analysis: The isolated circulating tumor biomarkers are then analyzed for the presence of cancer‐associated mutations, gene expression alterations, epigenetic changes, or specific protein markers. This analysis provides crucial information about the presence and characteristics of cancer cells.
- Data interpretation: The results obtained from the liquid biopsy analysis are interpreted by comparing them with known cancer‐associated biomarkers and genetic profiles. Clinicians use this information to assess the patient's cancer status, predict treatment response, monitor disease progression, and identify potential therapeutic targets.
- Validation and confirmation: The identified biomarkers are validated and confirmed to ensure accuracy and reliability. Validation may involve retesting samples, using alternative methods, or comparing results with traditional tissue biopsy data.

### 2.4 Range of cancers where liquid biopsy has utility

- Lung cancer: Liquid biopsy has been used to identify mutations in genes like EGFR, ALK, and ROS1, which are often targeted in the treatment of lung cancer.^18^, ^19^
- Ovarian cancer: Liquid biopsy has been investigated for detecting ovarian cancer‐associated mutations and monitoring disease progression.^32^
- Liver cancer: Liquid biopsy can be used to detect genetic mutations and biomarkers associated with hepatocellular carcinoma (HCC) and other liver cancers.^18^, ^19^
- Pancreatic cancer: This form of cancer can be extremely difficult to detect early and when detected late, it has very poor survival, thus the importance of screening for this cancer is critical and ctDNA,^25^ CTCs,^24^ EVs,^26^ and microRNA profiling^30^ have all been utilized.
- Lymphomas and leukemias: Liquid biopsy can be used to detect specific genetic alterations and monitor disease progression in various blood cancers.
- Nonsmall‐cell lung cancer (NSCLC): Liquid biopsy has been extensively studied for NSCLC patients due to the ease of accessing ctDNA in the bloodstream. It has been used to detect epidermal growth factor receptor (EGFR) mutations and monitor the emergence of resistance mutations during targeted therapy.^33^
- Colorectal cancer (CRC): Liquid biopsy has shown promise in detecting mutations in genes such as KRAS, NRAS, BRAF, and TP53 in CRC patients. It can be used for early detection, monitoring of treatment response, and identifying resistance mutations during targeted therapies.^34^
- Breast cancer: Liquid biopsy can help detect specific mutations, such as those in the BRCA1 and BRCA2 genes, which are associated with an increased risk of breast cancer. Liquid biopsy has been explored for monitoring treatment response and detecting mutations like HER2 amplifications and PIK3CA mutations in advanced breast cancer patients. Caputo et al.^35^ reported on the use of liquid biopsy in breast cancer.
- Prostate cancer: Urine analysis can detect prostate‐specific antigen (PSA) and genetic mutations associated with prostate cancer. Liquid biopsy has shown potential in monitoring treatment response and detecting androgen receptor (AR) mutations in patients with metastatic castration‐resistant prostate cancer (mCRPC).^36^

### 2.5 Use of liquid biopsy and NGS for general cancers and OSCC diagnostics

Liquid biopsy is a noninvasive diagnostic technique used to detect and monitor cancer by analyzing specific biomarkers present in bodily fluids, such as blood, urine, or cerebrospinal fluid. The analysis of these biomarkers provides valuable information about the presence of cancer, its genetic characteristics, and treatment response. Liquid biopsies are becoming increasingly important in cancer management due to their ability to offer real‐time and dynamic information, unlike traditional tissue biopsies, which can be invasive and limited to specific tumor locations. Liquid biopsy is a noninvasive diagnostic procedure used to detect and monitor cancers by analyzing various biomarkers present in bodily fluids, such as blood or saliva. This method provides an alternative to traditional tissue biopsies, which require invasive procedures to obtain a tissue sample directly from the tumor site. Liquid biopsy is particularly useful for detecting early stage cancers, monitoring treatment response, and detecting cancer recurrence.

For oral cancer, which includes cancers of the mouth, tongue, lips, and throat, liquid biopsy can be a valuable tool for early detection and monitoring of the disease's progression. The liquid biopsy for oral cancer primarily involves analyzing the biomarkers found in blood or saliva samples. As discussed previously the two main types of biomarkers examined in liquid biopsies are cell‐free nucleic acids (ctDNA) and circulating tumor cells (CTCs).

ctDNA are small fragments of tumor‐derived DNA that circulate in the bloodstream. Liquid biopsies can detect ctDNA and identify genetic mutations associated with cancer, even in early stages or during treatment. This technology has the potential to revolutionize cancer detection and monitoring. Tumors release these nucleic acids into bodily fluids due to cell death or apoptosis. By analyzing the genetic material present in these cell‐free nucleic acids, researchers can identify specific genetic mutations or alterations associated with oral cancer. This information can be used for early detection, monitoring treatment response, and tracking the development of resistance to therapies.^37^

CTCs are cancer cells that have detached from the primary tumor and entered the bloodstream or lymphatic system. They carry valuable information about the tumor's genetic makeup and can help identify the tumor's molecular characteristics. Detecting CTCs in blood can provide insight into the presence and aggressiveness of oral cancer and can assist in selecting appropriate treatment options.^38^

### 2.6 Monitoring treatment response and resistance

By analyzing ctDNA over time, liquid biopsies can provide insights into how tumors respond to treatment and the emergence of resistance mutations. This real‐time information enables clinicians to adjust treatment strategies accordingly.^20^

Liquid biopsy has utility as a noninvasive means of monitoring drug therapy and assessing the treatment response in cancer patients. With the analysis of various biomarkers such as CTCs, cell‐free DNA (cfDNA), exosomes, and ctDNA we can provide real‐time information about the tumor's genetic profile, response to treatment, and potential development of resistance, enabling more personalized and precise cancer management.^39^ Apart from ctDNA and CTCs, liquid biopsies can also analyze miRNAs and other biomolecules that are dysregulated in cancer. These markers can offer additional insights into tumor biology and disease progression.^40^ Dawson et al.^41^ provide a comprehensive review that covers the potential applications of liquid biopsies in cancer management, including monitoring drug therapy response, detecting minimal residual disease, and monitoring treatment resistance. The utility of ctDNA analysis in monitoring metastatic breast cancer patients undergoing treatment has been extensively considered and is of critical importance in the management of this disease with its likelihood of serious and negatively impactful recurrences. Researchers have found that changes in ctDNA levels correlate with the patient's treatment response and disease progression and thus can be used to tweak therapies and indicate prognosis and remission of the condition.^42^

Liquid biopsies hold promise for detecting minimal residual disease after surgery or other therapies. By monitoring residual tumor DNA in the bloodstream, clinicians can assess the risk of disease recurrence and tailor follow‐up treatment accordingly.^43^ Schwarzenbach et al.^44^ highlight the potential of liquid biopsy in detecting minimal residual disease in localized lung cancer patients after surgery, providing critical information for monitoring treatment efficacy and the risk of recurrence.

The various types of cell‐free nucleic acids, such as cfDNA and ctDNA, differ in their potential as critical biomarkers for cancer diagnosis, prognosis, and monitoring treatment response. These nuances are constantly being reviewed and our understanding improved and Diaz et al.^45^ provides insights into the molecular evolution of drug resistance in colorectal cancer patients through the analysis of ctDNA during a precision medicine type therapy.

Liquid biopsy has emerged as a promising tool in cancer management due to its noninvasive nature, potential for early detection of treatment response, and ability to track tumor evolution. As the field continues to advance, liquid biopsy is likely to play an increasingly important role in tailoring cancer treatment strategies and improving patient outcomes.

### 2.7 Liquid biopsy offers several advantages for oral cancer detection and management

- Noninvasiveness: Liquid biopsy is a minimally invasive procedure, requiring only a blood or saliva sample, making it more comfortable for patients and reducing potential risks and complications.
- Early Detection: Liquid biopsy can detect oral cancer at an early stage, even before the development of symptoms or visible tumors, increasing the chances of successful treatment.
- Monitoring Treatment Response: As oral cancer treatment progresses, liquid biopsy can be performed at regular intervals to monitor how the tumor responds to therapies. This helps doctors make timely adjustments to treatment plans if necessary.
- Detection of Recurrence: Liquid biopsy can be used to detect cancer recurrence earlier than traditional imaging techniques, allowing for prompt intervention and better patient outcomes.

However, it is essential to note that liquid biopsy is not a stand‐alone diagnostic tool. In some cases, a traditional tissue biopsy may still be required for definitive diagnosis and for determining the tumor's histological subtype. Additionally, while liquid biopsy is promising, it may not be as sensitive as tissue biopsy in certain situations. As with any diagnostic test, liquid biopsy results should be interpreted by healthcare professionals experienced in the field of oncology to ensure accurate diagnosis and appropriate treatment planning for oral cancer patients.

### 2.8 Liquid biopsy process and timeline

The time it takes to perform a liquid biopsy can vary depending on several factors, including the specific type of liquid biopsy test being conducted, the laboratory's processing time, and logistic considerations. Generally, the overall timeline can be broken down into different stages:

- Sample Collection: The actual collection of the blood or saliva sample for the liquid biopsy is a relatively quick procedure and typically takes only a few minutes. A healthcare professional performs the sample collection, for blood it is a standard venipuncture to draw the blood sample from the patient. In certain situations, a subject could collect their own saliva or perform a fingerprick blood sample, but the volume in most cases may be too low for proper analysis.
- Sample Transport: After the blood sample is collected, it needs to be properly labeled, entered into the laboratory and clinical digital system, packaged, and transported to the laboratory for analysis. The transportation time may vary depending on the location of the laboratory and the shipping method used.
- Sample Processing: Once the blood sample reaches the laboratory, the processing time can vary based on the specific type of liquid biopsy being performed and the laboratory's workload. In some cases, the sample may need to undergo initial centrifugation to separate the plasma or serum from the blood cells. DNA Extraction and Analysis: The isolation of cell‐free DNA (cfDNA) or circulating tumor DNA (ctDNA) from the blood sample is a critical step in the liquid biopsy process. Depending on the laboratory's protocols and the technology used, this step can take several hours to a few days. Isolation of Circulating Tumor Biomarkers: ctDNA, CTCs, exosomes, or other tumor‐specific biomarkers are isolated from the plasma or serum. Various techniques, such as PCR, digital PCR (dPCR), NGS, and microfluidics‐based platforms, can be used for this purpose.^20^
- Genetic Analysis and Interpretation: after the DNA extraction, the sample undergoes various genetic analysis techniques, such as NGS, PCR, or other molecular methods. The time for analysis and data interpretation can vary depending on the complexity of the test and the volume of data to be processed. The isolated circulating tumor biomarkers are then analyzed for the presence of cancer‐associated mutations, gene expression alterations, epigenetic changes, or specific protein markers. This analysis provides crucial information about the presence and characteristics of cancer cells.^20^, ^21^, ^22^, ^23^, ^46^
- Data Interpretation: The results obtained from the liquid biopsy analysis are interpreted by comparing them with known cancer‐associated biomarkers and genetic profiles. Clinicians use this information to assess the patient's cancer status, predict treatment response, monitor disease progression, and identify potential therapeutic targets.
- Reporting of Results: The identified biomarkers are validated and confirmed to ensure accuracy and reliability. Validation may involve retesting samples, using alternative methods, or comparing results with traditional tissue biopsy data. Once the analysis is completed, the laboratory generates a report summarizing the findings. The time taken to generate and deliver the report to the healthcare provider can vary, but it is usually within a few days to a couple of weeks.

Overall, the entire process, from sample collection to reporting of results, can take anywhere from a few days to a few weeks, depending on the specific circumstances. In some urgent cases, the laboratory may prioritize the processing and reporting of results to expedite the diagnostic process and aid in timely patient care. It is important to note that liquid biopsy technologies and laboratory practices are continually evolving, and the turnaround time for liquid biopsy tests may improve in the future with advances in technology and streamlined workflows.

### 2.9 Accuracy of liquid biopsy in cancer

The accuracy of liquid biopsy diagnosis for cancer has improved significantly in recent years but can vary depending on several factors. Overall, liquid biopsies have shown promise, especially for detecting certain types of mutations and monitoring treatment responses. However, as with all diagnostics, there are limitations and considerations associated with liquid biopsy accuracy:

- Sensitivity and specificity: Sensitivity refers to the test's ability to correctly identify patients with the disease (true positive rate), while specificity indicates the test's ability to correctly identify individuals without the disease (true negative rate). Liquid biopsies have generally shown high sensitivity for detecting cancer‐specific genetic mutations, but specificity can be affected by false positives due to the presence of mutations from noncancerous sources or other conditions.
  - Nonsmall‐cell lung cancer (NSCLC): A study published in JAMA Oncology in 2019 evaluated the performance of liquid biopsy using cell‐free DNA (cfDNA) in detecting EGFR mutations in NSCLC patients. The study showed a sensitivity of 68.4% and a specificity of 70.3% for detecting EGFR mutations using liquid biopsy when compared to tissue biopsy as the reference method.^33^
  - Colorectal cancer (CRC): Vacante et al.^47^ reviewed the multiple approaches, sampling sources, and the accuracy of liquid biopsy for detecting diagnostically relevant mutations in CRC patients. The sensitivity and specificity for detecting CRC were clinically useful for a range of diagnostic molecules and for a variety of sample sources.
  - Prostate cancer: A study published in European Urology in 2020 evaluated the performance of liquid biopsy in detecting genomic alterations in advanced prostate cancer patients. The study found a sensitivity of 94.6% and a specificity of 82.7% for detecting genomic alterations using liquid biopsy compared to tissue biopsy.^36^
- Tumor heterogeneity: The genetic profile of tumors can be diverse, with different regions of the tumor having distinct mutations. Liquid biopsies may not capture all genetic alterations present in the tumor, especially if the ctDNA released into the bloodstream is not representative of the entire tumor's genomic landscape.
- Detection threshold: Liquid biopsies rely on the presence of circulating tumor DNA (ctDNA) in the blood, which may be present in low concentrations, particularly in early stage cancers. The sensitivity of the test could be limited in cases where the ctDNA levels are too low for accurate detection.
- Tumor burden: Liquid biopsy accuracy can be influenced by the tumor's size and stage. Larger tumors typically release more ctDNA, making it easier to detect genetic mutations accurately.
- Technical challenges: The accuracy of liquid biopsies can be affected by technical factors, such as the isolation and detection methods used for ctDNA and other biomarkers. Advancements in technology and standardization of protocols have helped improve accuracy over time.
- False negatives: Although liquid biopsies can provide valuable diagnostic information, there is still a chance of false‐negative results, where the test fails to detect cancer or specific mutations. False negatives can occur due to technical limitations or insufficient ctDNA released into the bloodstream.

Despite these challenges, liquid biopsy has demonstrated its potential in various clinical applications, including cancer detection, treatment monitoring, and detecting resistance to targeted therapies. It is considered a useful tool, especially for screening but also when traditional tissue biopsies are challenging or impossible to perform, such as in cases where the tumor location is inaccessible or when patients cannot undergo invasive procedures.

### 2.10 Liquid biopsy accuracy in specific cancers

The question of accuracy for cancer detection is extensive and will not be covered in depth as it is outside the scope of this review, however, several illustrative examples are included here to attempt to characterize the state of the literature and the potential for this critical advance and its application to managing OSCC. It is essential to note that the accuracy may vary depending on the type and stage of cancer, the specific technology used for analysis, and the proficiency of the laboratory performing the test. As technology and research continue to advance, liquid biopsy techniques are likely to become even more accurate and widely adopted in cancer diagnosis and management. However, it is essential to interpret the results in conjunction with other clinical information and use them as complementary tools to improve patient care. Zhang et al.^48^ demonstrated the potential of urine‐based liquid biopsy using next‐generation sequencing to detect cancer‐associated mutations in patients with urological cancers. The sensitivity and specificity of the liquid biopsy method were evaluated against circulating tumor DNA (ctDNA) and using the gold standard of biopsy tissue DNA, showing highly promising sensitivity and specificity for urological cancer detection and good concordance across the blood ctDNA and urine (utDNA) derived free DNA.

Li et al.^49^ in 2018 reviewed state‐of‐the‐art of liquid biopsy analysis of circulating tumor DNA (ctDNA) and circulating tumor cells (CTCs) in patients with liver cancers. They demonstrated the accuracy of liquid biopsy in detecting liver cancer‐specific mutations and claimed it had potential as a noninvasive diagnostic tool for liver cancer detection. These approaches are now approved and being used by laboratories to support clinical management.

Mok et al.^10^ explored the utility of liquid biopsy in detecting EGFR mutations in the circulating tumor DNA of nonsmall‐cell lung cancer (NSCLC) patients. The findings indicated that liquid biopsy could predict survival outcomes in patients treated with erlotinib and chemotherapy, suggesting its potential as a prognostic and monitoring tool. This approach whereby NGS and liquid biopsy techniques are used to monitor chemotherapeutics and precision medicine approaches to cancer treatment is a considerably fast‐developing field and has the potential to finely and accurately tune therapies with tremendous benefits to cancer patients. Gattuso et al.^50^ reviewed various liquid biopsy technologies and their clinical utility in oncology. They discuss the accuracy and limitations of liquid biopsy for cancer detection and monitoring, taking into account different cancer types and stages and provide a useful assessment of the utility of this complex yet increasingly critical methodology in cancer management.

### 2.11 The potential practical use of liquid biopsy applications for oral squamous cell cancer diagnostics

Liquid biopsy is a noninvasive tool that potentially offers several applications for OSCC, including early detection, monitoring treatment response, and identifying potential therapeutic targets.

#### 2.11.1 Early detection of oral squamous cell carcinoma

Liquid biopsy can be utilized to identify circulating tumor DNA (ctDNA) or other cancer‐specific biomarkers in blood or saliva samples, enabling the early detection of OSCC. Egyud et al.^51^ explained the potential of ctDNA as a biomarker for OSCC detection. The study reported a high sensitivity and specificity in detecting OSCC cases using ctDNA analysis from saliva samples.

#### 2.11.2 Monitoring treatment response and disease progression

During the course of OSCC treatment, radiotherapy, chemotherapy or after surgical excision, liquid biopsy can be employed to monitor the dynamics of ctDNA and other biomarkers in the biofluids. A study by Hironaka et al.^52^ demonstrated that monitoring ctDNA levels in plasma could be used to assess treatment response and predict the risk of recurrence in OSCC patients undergoing surgery.

#### 2.11.3 Identification of therapeutic targets and drug resistance mechanisms

Liquid biopsy can also aid in the identification of specific genetic alterations and molecular pathways driving OSCC, which can potentially serve as therapeutic targets. Furthermore, it can help in understanding the mechanisms of drug resistance, leading to the development of more effective treatment strategies. A study by Trumet et al.^53^ in 2023 utilized liquid biopsy to identify genetic alterations associated with immune checkpoint inhibitor resistance in OSCC.

Thus, the current evidence suggests that liquid biopsy has significant applications in OSCC, ranging from early detection and treatment monitoring to identifying therapeutic targets and drug resistance mechanisms. However, it is worth noting that while liquid biopsy holds great promise, more research and validation are required to establish its widespread clinical utility in OSCC and other cancers.

Reis et al.^54^ reported on the use of four gene signatures to characterize surgical margins. While these signatures are based on tissues, it is feasible that such cell‐free or cell‐related DNA may be found in the bloodstream and could be detected using liquid biopsy and NGS techniques such that these signatures could be validated for detection of active OSCC. The four genes in question relate to MMP1, COL4A1, P4HA2, and THBS2, at least some of which are implicated in tissue turnover.

Fogel et al.^55^ reported on significant genes associated with cutaneous (CSCC), not oral squamous cell carcinoma, however, their findings may be applicable to OSCC. Similarly, Qui et al^56^ found that two genetic biomarkers, MYBL2 and TK1 are expressed higher in tissue with CSCC invasion groups compared with samples without invasion and these genes were the most significant biomarkers associated with CSCC initiation and progression. These genes positively correlate with N glycan biosynthesis and p53 signaling pathways and may prove to play a vital role in CSCC tumorigenesis and progression. Clearly, this data needs to be considered in OSCC pathology, but these genes may eventually, after clinical trials, be considered biomarkers for OSCC detection.

Bourova‐Flin et al.^57^ developed a specific gene expression signature/combination of three genes, AREG, CCNA1, and DDX20, which they found in silico to be associated with high‐risk OSCC subjects, and then translated these findings into a diagnostic test that was able to stratify high‐ and low‐risk patients. These genes have yet to be fully validated independently and shown to have clinical utility, but nonetheless, they may yet turn out to be valuable components of screening tests for genetic patients at risk of OSCC.

Elahi and Rakhshan^58^ performed an analysis of OSCC positive and adjacent negative biopsy tissue and found that surviving patients had upregulated tumoral expression of TGF‐β1 and downregulation of CD16, CD57, and MED15. These genes could be more relevant to local tissue activity and reflect this more than present as useful biomarkers that could be detected by liquid biopsy and NGS, but again this remains to be tested.

Arora et al.^59^ claimed, based on interrogating a cancer gene and clinical data repository, that NCBP2 and TFRC are novel prognostic biomarkers in oral squamous cell carcinoma, but much prospective work would be needed to affirm these hypotheses. As for the explosion of publications claiming associations between diseases and single nucleotide polymorphisms that occurred in the last 10 years, we can expect many putative associations to be made regarding gene signatures that could be diagnostic and/or therapeutic targets for OSCC, but randomized controlled clinical trials, hopefully, supported by mechanistic explanations, will be required to support these hypotheses. In a somewhat similar fashion to Arora et al.,^59^ Yang et al.^60^ had already reported that they had constructed a nine‐gene signature (TEX101, DSG2, SCG5, ADA, BOC, SCARA5, FST, SOCS1, and STC2) that could be associated with prognosis of OSCC and thus provide targets for immunotherapy. These gene targets need further validation of their potential clinical utility both for diagnosis and therapy.

### 2.12 How does liquid biopsy compare to traditional tissue biopsies in terms of accuracy?

Liquid biopsy and traditional tissue biopsy are two distinct diagnostic methods with their own strengths and limitations. Each method has its role and can complement the other in different clinical scenarios. However, it is useful to compare liquid biopsy and traditional tissue biopsy head‐to‐head in terms of accuracy:

- Accuracy in cancer detection:
  - Traditional tissue biopsy: Tissue biopsy involves directly obtaining a sample of the tumor or suspicious tissue for examination under a microscope. This method provides a definitive diagnosis with high accuracy as it allows pathologists to assess the tissue's cellular structure and identify cancerous cells.
  - Liquid biopsy: Liquid biopsy detects specific genetic mutations, epigenetic changes, and protein biomarkers associated with cancer through analysis of cell‐free DNA (cfDNA), circulating tumor DNA (ctDNA), and other biomolecules in the bloodstream. Liquid biopsy's accuracy for cancer detection has improved significantly, but it cannot always provide a complete picture of the tumor's histology and grading, especially in early stage cancers. The gold standard of histopathology will remain for the foreseeable future. In addition, histopathology will remain critical for checking excision margins, yet technology will continue to advance, and although precision imaging technologies in histopathology will keep pace, the ability and ease of liquid biopsy may prove decisive in the future. As already alluded to, the combination of both approaches should be most advantageous.
- Sensitivity and specificity:
  - Traditional tissue biopsy: Tissue biopsies generally have high sensitivity and specificity since they directly analyze the tumor tissue, allowing for a comprehensive assessment of cellular characteristics and genetic mutations, and will remain the gold standard.
  - Liquid biopsy: Liquid biopsies have high sensitivity for detecting cancer‐specific genetic mutations in the bloodstream. However, their specificity can be influenced by the presence of mutations from noncancerous sources or other conditions, leading to false‐positive results and probably more concerning, false negatives, where cancer is missed in some cases.
- Accessibility and invasiveness:
  - Traditional tissue biopsy: Tissue biopsy requires an invasive procedure to obtain a tissue sample, which may not be feasible or safe in certain cases, especially if the tumor is located in a hard‐to‐reach area.
  - Liquid biopsy: Liquid biopsy is a minimally invasive procedure that involves drawing a blood sample, making it a safer and more accessible option for patients who cannot undergo traditional tissue biopsies. In addition, the future approach with the aid of advances across the whole of the diagnostic field, may be that we can use liquid biopsy to screen and monitor regularly.
- Tumor heterogeneity:
  - Traditional tissue biopsy: Tissue biopsy allows for the assessment of tumor heterogeneity by examining multiple areas of the tumor, which can provide a more comprehensive understanding of the tumor's genetic makeup.
  - Liquid biopsy: Liquid biopsy may capture only a subset of the tumor's genetic mutations, which might not fully represent the tumor's heterogeneity, especially in cases where ctDNA levels are low.
- Monitoring treatment response and disease progression:
  - Traditional tissue biopsy: Tissue biopsy is not ideal for continuous monitoring of treatment response and disease progression as it involves repeated invasive procedures.
  - Liquid biopsy: Liquid biopsy is well suited for serial monitoring of treatment response and detecting emerging resistance to targeted therapies since it can be performed with a simple blood draw.

In summary, traditional tissue biopsy remains the gold standard for obtaining a definitive diagnosis and assessing tumor histology. Liquid biopsy complements traditional tissue biopsy by offering a minimally invasive option for cancer detection and monitoring, especially in situations where tissue biopsy is challenging or not possible. Both methods have their unique roles in cancer diagnosis and management, and their combined use can improve patient care and treatment decisions.

### 2.13 Liquid biopsy versus surgical biopsy for oral squamous cell cancer

#### 2.13.1 What are the limitations of traditional tissue biopsy?

Traditional tissue biopsy is a widely used diagnostic procedure for cancer and other diseases. However, it does have some limitations, which can impact its accuracy and utility in certain cases. Here are some of the key limitations of traditional tissue biopsy:

- Invasiveness and risk: Traditional tissue biopsy involves a surgical procedure or needle aspiration to obtain a tissue sample. This can be invasive, leading to discomfort, pain, and potential complications, such as bleeding or infection.^61^
- Sampling bias: Tissue biopsy provides a small sample of the tumor or affected tissue, which may not fully represent the entire tumor's heterogeneity. Sampling bias can lead to underestimation or mischaracterization of the tumor, potentially affecting treatment decisions.^61^
- Accessible tumors: In some cases, tumors may be located in anatomically difficult‐to‐reach areas or may be too small to be safely biopsied, making it challenging to obtain a tissue sample.

Serial monitoring: For some cancers or treatment monitoring purposes, multiple biopsies may be required to assess disease progression or treatment response. Repeated biopsies are not always feasible due to patient discomfort and risk.^62^, ^63^

- Timing and timing bias: The timing of the biopsy can influence the results, as tumors may evolve over time, and the sample may not reflect the current state of the disease. This is known as timing bias and can impact treatment decisions.
- Patient suitability: Certain patients, such as those with advanced disease or comorbidities, may not be suitable candidates for traditional tissue biopsy due to the associated risks and challenges.

These limitations highlight the need for complementary diagnostic approaches like liquid biopsy, which can help overcome some of the challenges associated with traditional tissue biopsy and provide valuable information for cancer diagnosis and monitoring. Liquid biopsy has been a growing area of research in the field of cancer diagnosis, including oral squamous cell carcinoma (OSCC).^50^

### 2.14 Liquid biopsy for OSCC

#### 2.14.1 Pros

- Noninvasive: Liquid biopsies can be easily collected from peripheral blood or saliva, reducing patient discomfort and risks associated with surgical procedures.
- Monitoring disease progression: Liquid biopsies can be used to monitor treatment response and detect recurrence earlier, allowing for timely interventions.
- Tumor heterogeneity: Liquid biopsies may capture a more comprehensive profile of tumor heterogeneity since they provide a systemic view of tumor‐derived materials.
- Real‐time and dynamic monitoring: Liquid biopsies enable serial sampling, allowing continuous monitoring of the tumor's genetic changes over time.

#### 2.14.2 Cons

- Sensitivity and specificity: Liquid biopsies may not always detect low levels of circulating tumor DNA, especially in early stage tumors, potentially leading to false‐negative results.
- Limited tissue information: Unlike surgical biopsies, liquid biopsies provide limited histological and morphological information about the tumor.

### 2.15 Surgical biopsy for OSCC

#### 2.15.1 Pros

- Accurate diagnosis: Surgical biopsies provide direct access to the tumor tissue, facilitating accurate diagnosis and characterization of the tumor's histopathological features.^64^
- Local staging: Surgical biopsies help in determining the extent of tumor involvement and regional lymph node status, which is crucial for treatment planning.
- Complete removal of tumor: In many cases, the surgical biopsy procedure can remove the entire tumor, providing a definitive treatment approach.

#### 2.15.2 Cons

- Invasive procedure: Surgical biopsies are invasive and may carry some risks, such as bleeding, infection, or damage to adjacent structures.
- Patient discomfort: The procedure can cause pain and discomfort for the patient.
- Delay in diagnosis: Surgical biopsies may require more time for tissue processing and pathological evaluation compared to liquid biopsies.

## 3 CONCLUSIONS AND FUTURE PERSPECTIVES

For oral squamous cell carcinoma, liquid biopsy is still in its infancy, but knowledge will expand such that it will be of great value in predicting susceptibility, early detection, monitoring disease progression and remission, response to therapy, and recurrence. This review has provided a basic introduction to this complex topic and hopefully will encourage greater research and adoption of this technology. OSCC is often detected late, but in future, a liquid biopsy may provide an effective screening process to detect OSCC in the early stages where prognosis is favorable and morbidity can be minimized.

### 3.1 Combining liquid biopsy approaches and big data in an AI approach to OSCC diagnostics

Clearly, the more information on patient risk factors related to a specific cancer the better the clinician can hone the diagnostic approach. For example, there is no benefit in doing a liquid biopsy for lung cancer in a subject who does not smoke or is not in an occupation (mining) or environment (high asbestos levels) where inhalation of carcinogens is common. Risk factors for oral cancer should thus be a critical addition to the consideration of the cost–benefit of a liquid biopsy screening approach and algorithms should be produced that combine both modalities to enhance diagnostic accuracy and utility. Several risk factors are associated with an increased likelihood of developing oral cancer. These risk factors include:

- Tobacco use: Smoking cigarettes, cigars, pipes, or using smokeless tobacco (chewing tobacco or snuff) increases the risk significantly. Tobacco contains harmful chemicals that can damage the cells in the mouth and lead to cancer.
- Alcohol consumption: Excessive and regular alcohol consumption, particularly when combined with tobacco use, can elevate the risk of developing oral cancer.
- Human Papillomavirus (HPV) infection: Certain strains of HPV, particularly HPV‐16 and HPV‐18, have been linked to an increased risk of oral cancer. HPV is primarily transmitted through sexual contact.
- Sun exposure: Prolonged and excessive exposure to sunlight without protection can lead to lip cancer.
- Age: The risk of oral cancer increases with age, especially after the age of 45.
- Gender: Men are more likely to develop oral cancer than women.
- Poor oral hygiene: Neglecting oral hygiene and failing to have regular dental checkups may increase the risk of developing oral cancer.
- Poor nutrition: A diet lacking in fruits and vegetables may increase the risk of oral cancer.
- Previous history of oral cancer: Individuals who have had oral cancer in the past have a higher risk of developing it again.
- Family history: Having a family history of oral cancer may increase the risk.
- Weakened immune system: People with weakened immune systems, such as those with HIV/AIDS or those who have undergone organ transplants, are at higher risk.

It is essential to note that while these risk factors increase the likelihood of developing oral cancer, in some cases, oral cancer can occur in individuals without any known risk factors. Like many types of cancer, oral cancer can result from a combination of genetic and environmental risk factors. While specific genetic factors contributing to oral cancer may vary among individuals and populations, several genes have been identified as potential risk factors: In oral cancer however, like lung cancer, while genetic factors can contribute to development, environmental risk factors prevail and lifestyle factors, such as tobacco and alcohol use, poor oral hygiene, human papillomavirus (HPV) infection, and exposure to UV radiation, play significant roles in oral cancer development. An argument can be made that it is the combination of both environmental and genetic risk factors that ultimately prevails in creating susceptibility, but more data is needed currently to make this conclusion.

Finally, it is important to stress that periodontists and hygienists have a clear role in detecting OSCC as they see patients with similar risk characteristics, such as smoking and poor oral hygiene, they see these patients at recall routinely, and they are adept at examining mucosa. Clearly, additional and continuous training in examining the floor of the mouth, palate, and cheeks and knowing what to consider suspicious as well as experience in seeing abnormal masses on radiographs is needed. In addition, a strong referral network that is interactive between periodontists and oral medicine and surgery colleagues is needed as biopsies and definitive diagnoses and treatment planning are complex. Nevertheless, we may see liquid biopsy migrate from a cancer specialist armamentarium to be used more routinely and even in a point‐of‐care setting in the years ahead. Overall, achieving the lofty goal of earlier OSCC diagnosis and reduced morbidity will include skilled primary screeners including periodontists and hygienists, and new diagnostic techniques such as liquid biopsy, and will ultimately save lives.

## Data Availability

No actual new data were used or referred to in this manuscript and as is typical of a review, all information referred to was previously published and is cited.
